# The prognostic value of serum erythropoietin in patients with lower-risk myelodysplastic syndromes: a review of the literature and expert opinion

**DOI:** 10.1007/s00277-019-03799-4

**Published:** 2019-10-25

**Authors:** Sophie Park, Charikleia Kelaidi, Mathieu Meunier, Nicole Casadevall, Aaron T. Gerds, Uwe Platzbecker

**Affiliations:** 1 CHU Grenoble, Université Grenoble Alpes, Institute for Advanced Biosciences, INSERM U1209, CNRS UMR 5309, CS 10217, 38043 Grenoble, France; 2 Aghia Sophia Children’s Hospital, Athens, Greece; 3 Hôpital Saint-Antoine, Paris, France; 4 Taussig Cancer Institute, Cleveland Clinic, Cleveland, OH USA; 5 Medical Clinic and Policlinic 1, Hematology and Cellular Therapy, Leipzig University Hospital, Leipzig, Germany

**Keywords:** Erythropoietin, Myelodysplastic syndromes, Prognosis, Erythropoiesis-stimulating agents, Hematologic response

## Abstract

Myelodysplastic syndromes (MDS) are hematopoietic stem cell malignancies associated with an erythroid maturation defect, resulting in anemia. Treatments for MDS include erythropoiesis-stimulating agents (ESAs). The identification of prognostic markers is important to help predict response and improve outcomes. Various scoring systems have been developed to help predict response to ESAs. Despite limitations in its assessment, serum erythropoietin (sEPO) level is an important predictor of hematologic response to ESAs in patients with lower-risk MDS. Numerous studies have reported significantly lower sEPO levels among responders versus non-responders. Furthermore, treatment response is significantly more likely among those with sEPO levels below versus those above various cutoffs. Other prognostic indicators for response to ESAs include lower transfusion requirement, fewer bone marrow blasts, higher hemoglobin, lower serum ferritin, lower-risk MDS, and more normal cytogenetics. Studies of other MDS therapies (e.g., lenalidomide and luspatercept) have also reported that lower sEPO levels are indicative of hematologic response. In addition, lower sEPO levels (up to 500 IU/L) have been included in treatment algorithms for patients with lower-risk MDS to define whether ESAs are indicated. Lower sEPO levels are predictive of hematologic response—particularly to ESAs. Further, clinical trials should use sEPO thresholds to ensure more homogeneous cohorts.

## Introduction

Myelodysplastic syndromes (MDS) are a heterogeneous group of hematopoietic stem cell malignancies that are characterized by an erythroid maturation defect, resulting in anemia, and can develop into acute myeloid leukemia (AML) [1, 2]. World Health Organization (WHO) MDS classifications from 2002 included refractory anemia (RA), refractory anemia with ring sideroblasts (RARS), refractory cytopenia with multilineage dysplasia (RCMD), RCMD and ring sideroblasts (RCMD-RS), refractory anemia with excess blasts (RAEB), and MDS associated with isolated del(5q) [3]. The most recent 2016 WHO MDS classifications are slightly different and include MDS with single lineage or multilineage dysplasia, MDS with ring sideroblasts and single or multilineage dysplasia, MDS with excess blasts, and MDS with isolated del(5q) [4].

The prognosis of patients with MDS, which is highly variable, can be assessed using various prognostic scoring systems. The earliest of these—the 1997 International Prognostic Scoring System (IPSS)—was largely based on the percentage of bone marrow blasts and karyotype [5]. The WHO classification–based Prognostic Scoring System (WPSS), which was introduced in 2007 [6], was largely based on the 2002 WHO MDS classifications [3] and karyotype. The recently revised 2012 IPSS (IPSS-R) [7] provides improved risk stratification over the original IPSS by incorporating more detailed cytogenetic subgroups and different thresholds for blast percentage and degree of cytopenias. These systems can all be used to categorize patients’ risks (e.g., low, intermediate, or high) in terms of survival and leukemic evolution.

A key first-line treatment option for many patients with symptomatic anemia associated with lower-risk MDS is erythropoiesis-stimulating agents (ESAs) (e.g., epoetin alpha or darbepoetin alpha), which may also be combined with granulocyte colony–stimulating factor (G-CSF) [8]. However, these are only recommended for patients with serum erythropoietin (sEPO) levels up to 500 IU/L [8]. In this review, we discuss the issues surrounding sEPO assessment and examine the available evidence for its prognostic value—primarily for response to ESAs—among patients with lower-risk MDS. We also discuss other prognostic markers for response to ESAs, and the inclusion of sEPO and other markers in various ESA response prediction scoring systems.

## Erythropoietin characterization

The concept of a hormone that regulates red blood cell (RBC) production was proposed in 1906 [9, 10] and, in 1948 [11], it was termed “erythropoietin” (EPO) [12]. EPO is mainly (90%) synthesized in peritubular cells in the kidney [13–15], with the remainder being produced in the liver [16]. Human EPO was first purified in 1977 [17] and the human EPO gene was cloned in 1985 [18, 19]. Recombinant human EPO (rhEPO) was shown to be comparable to natural EPO in 1986 [20] and, in 1987, synthetic EPO was first used to treat anemia associated with end-stage renal disease [21].

It is now well known that RBC production is regulated by EPO [12], which it does by binding to EPO receptors that are mainly expressed on immature erythroid cells [22, 23], thus stimulating their transformation into mature erythrocytes.

## sEPO assessment

Although sEPO assessment can be difficult due to its very low concentration in plasma (normally around 50 ng/L) [12], the first reliable bioassay was developed in 1955 [12, 24]. Originally, 1 unit of EPO was defined as the dose that gave the same erythropoiesis-stimulating response as 5-μmol cobaltous chloride [12]. In 1961, EPO standard A was produced (from sheep plasma), but this was quickly replaced by EPO standard B (from human urine) [25]. The international unit (IU) for EPO was then defined as the activity contained in 1.48 mg of EPO standard B [25]. The second international reference preparation of EPO was established in 1972 [26] and, in 1992, a purified recombinant deoxyribonucleic acid–derived human EPO was introduced [27].

A reliable radioimmunoassay for EPO was first developed in 1979 [12, 28] and enzyme-linked immunoassay kits are now used to measure EPO levels. Although currently available kits have good sensitivity (< 1 IU/L), their range generally only extends up to 100 IU/L [29–32] or 200 IU/L [33]. Although this is suitable for the general population, in whom sEPO levels are approximately 8 IU/L [34], they may not be able to accurately measure sEPO levels in MDS patients with symptomatic anemia, who can have highly elevated sEPO levels (> 10,000 IU/L [35]). Another potential complication when measuring sEPO levels is that they can vary throughout the day, although this is more significant in healthy individuals than in patients with MDS [36]. Accurate measurement of sEPO levels may also be complicated by the range of kits available, as there is likely some heterogeneity between them.

## Prognostic factors for response to ESAs in patients with MDS

**Fig. 1:**
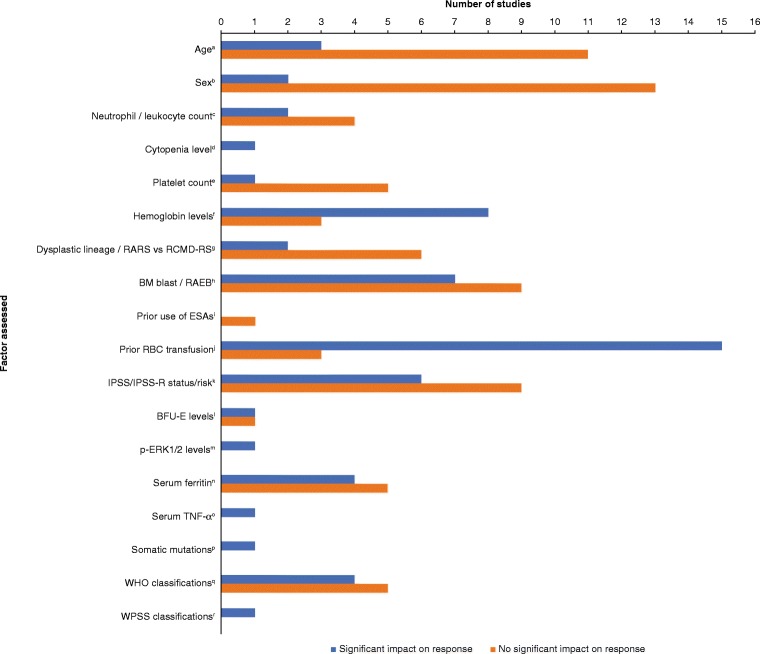
Non-sEPO factors predictive of hematologic response to ESAs in patients with MDS (mainly lower-risk). *ANC* absolute neutrophil count, *BFU-E* burst-forming unit-erythroid, *BM* bone marrow, *EPO* erythropoietin, *ESA* erythropoiesis-stimulating agent, *IPSS* International Prognostic Scoring System, *IPSS-R* revised International Prognostic Scoring System, *MDS* myelodysplastic syndromes, *RAEB* refractory anemia with excess blasts, *RARS* refractory anemia with ring sideroblasts, *RBC* red blood cell, *RCMS-RS* refractory cytopenia with multilineage dysplasia and ring sideroblasts, *sEPO* serum erythropoietin, *TNF-α* tumor necrosis factor alpha, *WHO* World Health Organization, *WPSS* WHO classification-based Prognostic Scoring System. (a) Hellstrom-Lindberg [39], Hellstrom-Lindberg [40], Stasi [44], Park [47], Gotlib [48], Greenberg [49], Frisan [50], Westers [51], Park [52], Santini [56], Kosmider [59], Houston [61], Park [62]; (b) Hellstrom-Lindberg [39], Hellstrom-Lindberg [42], Stasi [44], Mannone [45], Park [47], Gotlib [48], Greenberg [49], Frisan [50], Park [52], Santini [56], Kosmider [59], Buckstein [60], Houston [61], Park [62], Remacha [63]; (c) Hellstrom-Lindberg [39], Hellstrom-Lindberg [40], Stasi [44], Santini [56], Buckstein [60], Remacha [63], Howe [64]; (d) Molteni [57]; (e) Hellstrom-Lindberg [39], Hellstrom-Lindberg [40], Stasi [44], Santini [56], Buckstein [60], Remacha [63]; (f) Hellstrom-Lindberg [39], Hellstrom-Lindberg [40], Stasi [44], Frisan [50], Westers [51], Park [52], Kelaidi [54], Santini [56], Buckstein [60], Houston [61], Park [62]; (g) Mannone [45], Park [47], Greenberg [49], Park [52], Molteni [57], Kosmider [59], Remacha [63]; (h) Hellstrom-Lindberg [39], Hellstrom-Lindberg [40], Wallvik [41], Hellstrom-Lindberg [42], Musto [43], Stasi [44], Mannone [45], Park [47], Greenberg [49], Frisan [50], Westers [51], Park [52], Kelaidi [54], Santini [56], Molteni [57], Houston [61]; (i) Gabrilove [46]; (j) Hellstrom-Lindberg [39], Hellstrom-Lindberg [40], Wallvik [41], Hellstrom-Lindberg [42], Musto [43], Stasi [44], Mannone [45], Park [47], Gotlib [48], Greenberg [49], Frisan [50], Westers [51], Kelaidi [54], Santini [56], Molteni [57], Kosmider [59], Buckstein [60], Houston [61], Howe [64]; (k) Hellstrom-Lindberg [42], Stasi [44], Mannone [45], Park [47], Gotlib [48], Greenberg [49], Frisan [50], Westers [51], Park [52], Kelaidi [54], Santini [56], Kosmider [59], Buckstein [60], Houston [61], Park [62]; (l) Stasi [44], Frisan [50]; (m) Frisan [50]; (n) Hellstrom-Lindberg [40], Westers [51], Park [52], Kelaidi [54], Santini [56], Kosmider [59], Buckstein [60], Houston [61], Park [62]; (o) Stasi [65]; (p) Kosmider [59]; (q) Mannone [45], Park [47], Frisan [50], Westers [51], Park [52], Kelaidi [54], Santini [56], Kosmider [59], Park [62]; (r) Westers [51]

In general, factors that can be used to predict response to treatments can be used to tailor treatments more efficiently and, hence, improve outcomes. Many studies have examined factors that are prognostic for response to ESAs (with or without G-CSF) among patients with lower-risk MDS, and these are discussed below. Of note, response definitions were generally based on International Working Group (IWG) hematologic improvement criteria from 2000 [37] or 2006 [38], but this varied by study (as detailed in Table 1 [39–62] and Fig. 1 [39–52, 54, 56, 57, 59–65]).

**Table 1:** sEPO levels predictive of hematologic response to ESAs in patients with MDS (mainly lower-risk)

Reference	Treatment	*n*	Response definition	sEPO responders vs non-responders, IU/L^a^	sEPO cutoffs, IU/L	Response by sEPO, %
Hellstrom-Lindberg [39]	EPO + G-CSF	98	Hb ≥ 115 g/L or Hb ↑ ≥ 15 g/L or 100% reduction in transfusion need and stable Hb for ≥ 6 weeks	118 (range 6–1144) vs 741 (range 8–5921) (*P* < 0.001)	≤ 100 vs > 100	64 vs 26 (*P* < 0.001)
					≤ 500 vs > 500	55 vs 10 (*P* < 0.001)
Hellstrom-Lindberg [40]	EPO + G-CSF	71	Hb ≥ 115 g/L or Hb ↑ ≥ 15 g/L (non-transfusion patients) or 100% reduction in transfusion need and stable Hb for ≥ 4 weeks (transfusion patients)	247 ± 318 vs 1293 ± 1531 (*P* = 0.008)	< 100 vs ≥ 100	50 vs 29 (*P* = NS)
					< 500 vs ≥ 500	48 vs 16 (*P* = 0.02)
Wallvik [41]	EPO	68	Hb ↑ ≥ 15 g/L (non-transfusion patients) or elimination of transfusion need for ≥ 6 weeks (transfusion patients)	85 ± 74 vs 427 ± 464 (*P*_uni_ = 0.001, *P*_multi_ = 0.009)	NR	NR
Hellstrom-Lindberg [42]	EPO + G-CSF	53	Hb ≥ 115 g/L or Hb ↑ ≥ 15 g/L (non-transfusion patients) or 100% reduction in transfusion need and stable Hb for ≥ 4 weeks (transfusion patients)	NR	< 100 vs ≥ 100	56 vs 22 (*P* = 0.02)
					< 200 vs ≥ 200	45 vs 18 (*P* = NS)
					100–200 vs 200–500	25 vs 25 (*P* = NS)
Musto [43]	Darbepoetin alpha	37	IWG 2000^b^	NR	< 100 vs ≥ 100	65 vs 12 (*P* < 0.003)
Stasi [44]	Darbepoetin alpha	53	IWG 2000^b^	97 (range 26–370) vs 275 (56–515) (*P* < 0.001)	NR	NR
Mannone [45]	Darbepoetin alpha	62	IWG 2000^b^	NR	< 100 vs > 100	86 vs 58 (*P* = 0.013)
					< 200 vs > 200	82 vs 53 (*P* = 0.032)
Gabrilove [46]	Darbepoetin alpha	206	IWG 2006^c^	NR	< 100 vs 100–< 500 vs ≥ 500	51 vs 35 vs 19 (*P* = NR)
Park [47]	EPO ± G-CSF	403	IWG 2000^b^	NR	≤ 200 vs > 200	69 vs 42 (*P*_uni_ < 0.001, *P*_multi_ = 0.03)
Gotlib [48]	Darbepoetin alpha ± G-CSF	24	IWG 2000^b^	102 (range 12–422) vs 178 (range 44–2556) (*P* = 0.06)	<150 vs ≥150	81 vs 38 (*P* = 0.06)
Greenberg [49]	EPO ± G-CSF	73	IWG 2006^c^ (but response had to be sustained for ≥ 4 months)	40 (range 9–638) vs 142 (range 22–5466) (*P* = NR)	< 200 vs > 200	45 vs 5 (*P* = 0.002)
Frisan [50]	ESA ± G-CSF	127	IWG 2006^c^	35 (IQR 17–98) vs 122 (IQR 45–234) (*P* = 0.005)	< 100 vs ≥ 100	72 vs 42 (*P* = 0.006)
Westers [51]	EPO ± G-CSF	46	IWG 2006^c^	76 (range 19–587) vs 187 (range 33–6000) (*P* = 0.001)	< 100 vs > 100	71 vs 12 (*P* = NR)
Park [52]	EPO ± G-CSF	112	IWG 2006^c^	NR	≤ 100 vs 100–500	72 vs 30 (*P*_uni_ = 0.0003; *P*_multi_ = 0.02)
Villegas [53]	Darbepoetin alpha ± G-CSF	44	IWG 2000^b^	NR	< 100 vs ≥ 100	80 vs 26 (*P* = 0.0003)
Kelaidi [54]	Darbepoetin alpha ± G-CSF	99	IWG 2006^c^	NR	< 100 vs ≥ 100	66 vs 21 (*P* < 0.0001)
Kelaidi [55]	ESA ± G-CSF	253	IWG 2006^c^	33 (IQR 19–66) vs 53 (IQR 31–145) vs 104 (IQR 46–238) (*P* = 0.02)^d^	NR	NR
Santini [56]	ESA	456	IWG 2006^c^	NR	≤ 100 vs > 100	75 vs 45 (*P* < 0.0002)
					≤ 200 vs > 200	75 vs 31 (*P* < 0.0001)
Molteni [57]	EPO	58	IWG 2006^c^	NR	≤ 80 vs > 80	OR = 0.10; 95% CI, 0.03–0.35 (*P*_multi_ < 0.0005)
					≤ 100 vs > 100	OR = 0.16; 95% CI, 0.05–0.54 (*P*_multi_ = 0.003)
Jang [58]	Darbepoietin alpha	50	IWG 2000^b^	NR	< 100 vs ≥ 100	93 vs 44 (*P* = NR)
					< 200 vs ≥ 200	82 vs 39 (*P* = NR)
					< 300 vs ≥ 300	62 vs 50 (*P* = NR)
Kosmider [59]	ESA	79	IWG 2006^c^	NR	< 100 vs > 100	76 vs 39 (*P*_uni_ = 0.002; *P*_multi_ = 0.04)
Buckstein [60]	ESA ± G-CSF	996	IWG 2006^c^	87 ± 194 vs 208 ± 332 (*P* < 0.0001)	< 100 vs ≥ 100	OR = 2.3 (*P* = 0.001)
Houston [61]	ESA ± G-CSF	208	IWG 2006^c^	NR	< 100 vs ≥ 100	OR_uni_ = 8.3 (*P*_uni_ < 0.0001); OR_multi_ = 8.7 (*P*_multi_ < 0.0001)
					< 200 vs ≥ 200	OR_uni_ = 4.9 (*P*_uni_ = 0.007)
Park [62]	ESA ± G-CSF	1698	IWG 2006^c^	60 (IQR 21–75) vs 183 (IQR 38–323) vs 245 (IQR 49–260) (*P* < 0.001)^d^	NR	NR

*CI* confidence interval, *EPO* erythropoietin, *ESA* erythropoiesis-stimulating agent, *G-CSF* granulocyte colony–stimulating factor, *Hb* hemoglobin, *IQR* interquartile range, *IU* international unit, *IWG* International Working Group, *MDS* myelodysplastic syndromes, *NR* not reported, *NS* not significant, *OR* odds ratio, *OR*_*multi*_ odds ratio by multivariable analysis, *OR*_*uni*_ odds ratio by univariate analysis, *P*_*multi*_*P* value by multivariable analysis, *P*_*uni*_*P* value by univariate analysis, *RBC* red blood cell, *SD* standard deviation, *sEPO* serum erythropoietin

^a^Values are mean ± SD or median (range or IQR) for responders versus non-responders

^b^IWG 2000 response criteria: for patients with pretreatment Hb < 110 g/L, ≥ 10 g/L increase in Hb; for RBC transfusion-dependent patients, 50% decrease in transfusion requirements. Responses have to last ≥ 2 months [38]

^c^IWG 2006 response criteria: for patients with pretreatment Hb < 110 g/L, ≥ 15 g/L increase in Hb; reduction of ≥ 4 RBC transfusions/8 weeks versus pretreatment 8 weeks (only RBC transfusions for a Hb ≤ 9.0 g/dL). Responses have to last ≥ 8 weeks [39]

^d^In these studies, sEPO levels were reported for patients who responded and did not relapse versus those who responded and relapsed versus those with primary resistance to ESAs

### sEPO levels and response to ESAs

Multiple studies have reported correlations between sEPO levels and response to ESAs with or without G-CSF among predominantly lower-risk MDS patients (Table 1) [39–62]. Nearly all the studies listed in Table 1 reported response rates to ESAs with or without G-CSF among patients with sEPO levels below versus above various cutoff levels. The most commonly reported sEPO cutoff was 100 IU/L, for which reported response rates were 50–93% for patients with sEPO < 100 IU/L versus 12–58% for patients with sEPO > 100 IU/L. Using a sEPO cutoff of 200 IU/L, 45–82% of patients with sEPO below the cutoff versus 5–53% of patients with higher sEPO levels responded; for a cutoff of 500 IU/L, 48–55% versus 10–16% responded, respectively. Most response comparisons by above versus below sEPO cutoff were significant.

Among studies that reported mean or median sEPO levels among responders versus non-responders, all reported lower sEPO levels among responders, although the actual values varied widely between studies (Table 1). The sEPO differences were significant in all but two studies: significance was not reported in one study [49] and one study only included 24 patients [49]. Two studies classified patients as having primary resistance to ESAs, relapsing after an initial response, or continuing to respond [55, 62]. Median sEPO levels in these three groups decreased significantly from resistant to relapsing to responding (Table 1) [55, 62].

Of note, among studies that reported ranges of sEPO levels among responders and non-responders, patients with an sEPO level as high as 1144 IU/L responded, while those with an sEPO level as low as 8 IU/L did not respond (Table 1), suggesting that sEPO levels cannot be guaranteed to predict response. Hence, additional prognostic indicators are needed.

### Other factors predictive of response to ESAs

A wide range of other markers have been correlated with improved hematologic response to ESAs with or without G-CSF among patients with lower-risk MDS (Fig. 1) [39–52, 54, 56, 57, 59–65]. The most commonly reported non-sEPO markers for improved response are lower transfusion requirement and higher hemoglobin level. Other commonly cited markers include fewer bone marrow blasts (lower percentage or RA/RARS rather than RAEB), lower serum ferritin level, lower-risk MDS using various prognostic schemes (e.g., IPSS, WPSS, IPSS-R), and more normal cytogenetics (e.g., lower-risk IPSS-R karyotypes). There have also been reports that lower tumor necrosis factor alpha level, being ESA-naïve, and shorter time to ESA onset are associated with improved response. A recent meta-analysis of darbepoetin alpha in MDS [66] has similarly reported that being ESA-naïve and having higher baseline hemoglobin level, higher dose, transfusion independence, and low-risk IPSS—along with sEPO level below 100 IU/L—are all linked with improved response rates.

It should be noted that many of the abovementioned factors that have been correlated with improved response to ESAs have also been correlated with improved prognosis [5–7]. It is therefore possible that patients with less aggressive disease are more likely to respond to ESAs, so these markers (including sEPO) may actually be predictors of disease severity rather than merely response to ESAs [67].

One study has examined the effect of somatic mutations on erythroid response [59]. In univariate analysis, having more than two mutations reduced the likelihood of response (odds ratio [OR], 0.29; 95% confidence interval [CI], 0.11–0.78; *P* = 0.01) compared with fewer mutations, but this was no longer significant in multivariate analysis. Higher numbers of mutations were, however, correlated with worse overall survival (hazard ratio [HR], 2.53; 95% CI, 1.00–7.20; *P* = 0.05), which suggests that alternative treatments may be required in such patients.

## Scoring systems predictive of hematologic response to ESAs among patients with MDS

Based on the most influential predictive factors discussed above, various groups have proposed scoring systems to predict hematologic response to ESAs with or without G-CSF among patients with MDS (Table 2) [39, 56, 60, 61]. These all include sEPO levels, although the cutoffs and scores vary between systems. The earliest system additionally included only transfusion need [39]. Later systems [56, 60, 61] included either IPSS-R or IPSS risk levels. Response rates for the most favorable scores are 74–85%, falling to 7–23% for the least favorable scores (Table 2).

**Table 2:** Scoring systems for predicting hematologic response to ESAs in patients with MDS (mainly lower-risk)

	Nordic (1997) (Hellstrom-Lindberg [39])	European (2013) (Santini [56])	MDS-CAN ESA (2017) (Houston [61])	ITACA (2017) (Buckstein [60])
Predictive factor (score adjustment)				
sEPO, IU/L	< 100 (+ 2) 100–500 (+ 1) > 500 (− 3)	> 200 (+ 1)	< 100 (+ 2)	< 100 (+ 1)
RBC transfusion need, units/month	< 2 (+2) ≥ 2 (− 2)	–	–	0 (+ 1)
IPSS	–	–	Low (+1)	Low (+ 1)
IPSS-R		Low (+ 1) Int (+ 2) High (+ 3)	–	–
Serum ferritin, ng/mL	–	> 350 (+ 1)	–	–
Predictive scores (% of patients achieving a response)				
Best to worst	> 1 (74) ± 1 (23) <− 1 (7) (*P* = NR)	0 (85) 1 (80) 2 (64) 3 (40) 4 (20) (*P* = NR)	3 (81) 2 (55) 1 (30) 0 (17) (*P* < 0.0001)	3 (85) 2 (67) 1 (43) 0 (23) (*P* < 0.0001)

*ESA* erythropoiesis-stimulating agent, *Int* intermediate, *IPSS* International Prognostic Scoring System, *IPSS-R* revised International Prognostic Scoring System, *ITACA* Italy-Canada, *IU* international unit, *MDS* myelodysplastic syndromes, *MDS-CAN ESA* myelodysplastic syndromes-Canada erythropoiesis–stimulating agent, *NR* not reported, *sEPO* serum erythropoietin

Various groups have corroborated that the Nordic score [39] is predictive of response to ESAs. Remacha et al. [63] reported response rates to rhEPO with or without G-CSF of 78% versus 15% for scores of > 1 versus ± 1 (*P*_uni_ = 0.0001; risk ratio [RR], 11.6; 95% CI, 2.5–53; *P*_multi_ = 0.0016) among 32 patients [63]. Hellstrom-Lindberg et al. [42] later validated their own score in 53 patients, showing that 61% versus 14% of those with scores of > 1 versus ± 1 responded (*P* = 0.001). Similarly, Molteni et al. [57] reported that 64% versus 33% of those with scores of > 1 versus ± 1 responded (*P* = 0.05). However, they also reported that 79% versus 33% of patients with scores of 4 versus 3 responded (*P* = 0.004), showing that those with a score of 3 had the same response rate as those with a score of ± 1.

Houston et al. [61], who designed the myelodysplastic syndromes-Canada ESA (MDS-CAN ESA) score, also tested the Nordic [39] and European [56] scores. Using the Nordic score, they found that 57% versus 31% of patients with scores of > 1 versus ± 1 responded (*P* = 0.01) [61]. They reported a non-significant declining trend of response rate to ESAs (67% vs 58% vs 52% vs 40% vs 13%) with increasing European scores. However, the lack of significance was likely due to a lack of power as all the required variables (ferritin, sEPO, and IPSS-R) were only available in 92 patients. Buckstein et al. [60], who designed the Italy-Canada (ITACA) score, also tested the Nordic, European, and MDS-CAN ESA scores (Table 2), but in much larger numbers of patients (*n* = 846, 524, and 702, respectively). They reported response rates of 68–78% for the best categories, falling to 20–38% for the worst categories for these three scores, suggesting that all of them could be very beneficial in predicting response to ESAs.

## Inclusion of sEPO assessments in MDS treatment guidelines

The importance of the predictive value of sEPO for response to ESAs is corroborated by its inclusion as a deciding factor for treatment in various MDS guidelines. For example, for patients with lower-risk MDS and symptomatic anemia, the current National Comprehensive Cancer Network Clinical Practice Guidelines in Oncology (NCCN guidelines®) for Myelodysplastic Syndromes depend on the presence/absence of the del(5q) cytogenetic abnormality and sEPO level [8]. For patients with del(5q), lenalidomide is indicated; for patients without del(5q), ESAs (epoetin alpha or darbepoetin alpha) are only recommended for those patients with sEPO levels up to 500 IU/L; for patients without del(5q) and sEPO levels above 500 IU/L, biologic response modifiers, hypomethylating agents, or clinical trials are indicated [8]. Similarly, the American Society of Hematology [68] only recommends ESAs for patients with lower-risk MDS and symptomatic anemia if they have sEPO levels below 500 IU/L.

The recommended sEPO level cutoff of 500 IU/L seems quite high, given that most studies and prediction scores use cutoffs of 100 or 200 IU/L (Tables 1 and 2). Also, many kits used to assess sEPO levels have detection limits of 100 IU/L [29–32] or 200 IU/L [33]. Further, sEPO levels can vary depending on factors such as hemoglobin level, time since last transfusion, and time of day. Therefore, a level of 500 IU/L was likely chosen for the guidelines to avoid having to deny ESAs to patients who might still respond to them. Of course, rather than relying solely on an sEPO level below 500 IU/L, it may be beneficial to use one of the predictive scoring systems described in Table 2 to further ascertain the likelihood of response to ESAs.

Based on three large studies of mainly lower-risk MDS patients treated with ESAs with or without G-CSF, approximately 80% of patients have sEPO levels below 200 IU/L [47, 56] and only around 10% of patients have sEPO levels above 500 IU/L [46]. Therefore, approximately 90% of lower-risk MDS patients would be eligible to receive ESAs according to guidelines. Given that only around 10–20% of patients with sEPO levels above 500 IU/L would likely respond to ESAs [39, 40, 46], such patients are recommended to receive alternative treatments (e.g., biologic response modifiers or hypomethylating agents) [8]. It should be noted that patients with sEPO levels of 200–500 IU/L have a lower chance of response than those with sEPO levels below 200 IU/L (see Table 1) and are thus more likely to require additional/alternative treatments. In patients with no response to ESAs with or without G-CSF after 3 months (or if response is lost), alternative treatments (e.g., lenalidomide) are recommended [8].

## Additional prognostic uses of sEPO among patients with MDS

sEPO levels have not only been correlated with response to ESAs. Various studies have also examined whether sEPO levels affect duration of response [44, 47, 59] and overall survival [41, 49, 55] among patients treated with ESAs. Other studies have examined whether sEPO levels can affect response to non-ESA treatments [69–72], or the effect of sEPO on progression to AML and overall survival among patients with de novo MDS [73].

### Duration of response in patients treated with ESAs

Three studies were identified that reported duration of response to ESAs with or without G-CSF by sEPO level [44, 47, 59]. Stasi et al. [44] reported response durations by sEPO level among 24 responders to darbepoetin alpha. Although no statistical analyses were performed, there did appear to be some correlation between longer survival and lower sEPO. However, Kosmider et al. [59] reported that sEPO level was not significantly correlated with response duration among 79 patients treated with an ESA with or without G-CSF. Similarly, in a study of 403 patients who received EPO with or without G-CSF, Park et al. [47] found that there was no significant difference in duration of response using a cutoff of 200 IU/L (20 months among patients with sEPO levels below 200 IU/L; 25 months among those with sEPO levels ≥ 200 IU/L; HR, 1.0; 95% CI, 0.6–1.7; *P* = 0.97).

### Overall survival in patients treated with ESAs

Various studies have reported on overall survival among patients treated with ESAs by sEPO level, but with inconsistent findings. In a study of 66 patients treated with EPO, Wallvik et al. [41] reported that median survival generally increased with decreasing sEPO level (e.g., 25 months for those with sEPO > 200 IU/L; 28 months for ≤ 200 IU/L; 38 months for ≤ 100 IU/L; and 65 months for ≤ 50 IU/mL). However, this was only significant for the 50, 70, and 100 IU/L cutoffs, but not for the 40, 150, or 200 IU/L cutoffs. Similarly, among 53 patients treated with EPO, survival was better among those with sEPO levels lower than 200 IU/L (34.2%) than those with 200 IU/L and higher (13.3%), after a median follow-up of 5.8 years [49]. In a study that categorized patients as relapsed after ESAs (*n* = 66) or refractory to ESAs (*n* = 120), survival decreased with lower sEPO, but only among those who responded and then relapsed (median survival 30.7 months [sEPO ≤100 IU/L] vs not reached [sEPO >100 IU/L]; HR, 0.38; 95% CI, 0.15–0.94; *P* = 0.036) [55]. Among patients with refractory MDS, the difference was not significant (median survival 38.6 months [sEPO ≤ 100 IU/L] vs 50.8 months [sEPO > 100 IU/L]; HR, 0.88; 95% CI, 0.59–1.33; *P* = 0.56) [55]. The mechanism underlying the inverse relationship between sEPO and overall survival is not fully understood but may involve a combination of factors. Resistance to endogenous EPO, a predictor of poor outcome [55, 62], can result in elevated sEPO levels. High EPO level, therefore, could be a marker of more aggressive disease that defines a population of patients with poor prognosis.

### Prediction of response to non-ESA treatments

To our knowledge, three studies have reported on erythroid response to lenalidomide with or without EPO in transfusion-dependent, ESA-refractory/ineligible patients with non-del(5q) lower-risk MDS (Table 3) [69–71]. Santini et al. [69] reported significantly better responses in patients with sEPO ≤ 500 IU/L versus > 500 IU/L by univariate analysis, but not by multivariate analysis. They also reported a significant trend for response by various cutoffs (Table 3). In a study by Toma et al. [70], an sEPO cutoff of 100 IU/L was predictive of response in univariate (OR, 3.3; 95% CI, 1.4–7.9; *P* = 0.009) and multivariate (OR, 4.1; 95%, CI 1.3–12.6; *P* = 0.02) analyses. In a smaller study, lower mean sEPO levels were reported among responders versus non-responders, but this was not significant [71].

**Table 3:** sEPO levels predictive of erythroid response to treatments other than ESAs among patients with MDS

Reference	Treatment; patients	*n*	Response definition	sEPO responders vs non-responders, IU/L^a^	sEPO cutoffs, IU/L	Erythroid response by sEPO, %
Santini [69]	Lenalidomide; TD, ineligible/refractory to ESAs, non-del(5q)	160	Transfusion independence for ≥ 8 weeks	NR	≤ 500 vs > 500	34 vs 16 (*P*_uni_ = 0.015; *P*_multi_ = NS)
					≤ 100 vs 100–200 vs 200–500 vs > 500	43 vs 33 vs 23 vs 16 (*P*_trend_ = 0.002)
Toma [70]	Lenalidomide ± EPO; TD, ESA-refractory, non-del(5q)	131	IWG 2006^b^	NR	< 100 vs ≥ 100	47 vs 21 (*P*_uni_ = 0.0087; *P*_multi_ = 0.016)
Komrokji [71]	Lenalidomide; TD, failed rhEPO, non-del(5q)	32	IWG 2000^c^	255 ± 283 vs 870 ± 1298 (*P* = NS)	NR	NR
Platzbecker [72]	Luspatercept; TD, mainly failed ESAs	58	IWG 2006^b^	NR	< 200 vs 200–500 vs ≥ 500	76 vs 58 vs 43 (*P* = NR)
					< 100 vs ≥ 100	EC_uni_ = 1·55 (*P*_uni_ = 0.03); EC_multi_ = 1·71 (*P*_multi_ = 0.04)

*EC*_*multi*_ estimated coefficient by multivariate analysis, *EC*_*uni*_ estimated coefficient by univariate analysis, *EPO* erythropoietin, *ESA* erythropoiesis-stimulating agent, *IWG* International Working Group, *MDS* myelodysplastic syndromes, *NR* not reported, *NS* not significant, *P*_*multi*_*P* value by multivariable analysis, *P*_*trend*_*P* value for the trend, *P*_*uni*_*P* value by univariate analysis, *rhEPO* recombinant human erythropoietin, *SD* standard deviation, *sEPO* serum erythropoietin, *TD* transfusion dependent

^a^Values are mean ± SD for responders versus non-responders

^b^IWG 2006 response criteria: for patients with pretreatment Hb < 110 g/L, ≥ 15 g/L increase in Hb; reduction of ≥ 4 RBC transfusions/8 weeks versus pretreatment 8 weeks (only RBC transfusions for a Hb ≤ 9.0 g/dL). Responses have to last ≥ 8 weeks [38]

^c^IWG 2000 response criteria: for patients with pretreatment Hb < 110 g/L, ≥ 10 g/L increase in Hb; for RBC transfusion-dependent patients, 50% decrease in transfusion requirements. Responses have to last ≥ 2 months [39]

Erythroid response in MDS patients receiving luspatercept has also been reported to vary by sEPO level (76% for sEPO < 200 IU/L; 58% for 200–500 IU/L; 43% for > 500 IU/L) [72]. Using a cutoff of 100 IU/L, sEPO had a significant effect on response in univariate and multivariate analyses (details in Table 3).

### Prediction of progression to AML and overall survival in patients with de novo MDS

Cortesao et al. [73] examined the effect of sEPO level on progression to AML and overall survival in a study of 102 patients with de novo MDS. They found that patients who developed AML had higher mean sEPO levels than those who did not (*P* < 0.05) and that a sEPO level above 57 IU/L had an influence on progression. The authors also reported that overall survival increased with decreasing sEPO levels (*P* = 0.03).

A higher sEPO level is associated with a low probability of response to ESAs [47], and it has been suggested that failure of ESA therapy is a marker of poor prognosis in patients with lower-risk MDS [55, 62]. The relationship between response to ESAs and the incidence of AML has been evaluated in several studies. In a study involving 253 patients with non-del(5q) lower-risk MDS who failed ESA therapy, the 5-year cumulative incidence of AML was significantly higher in patients experiencing early ESA failure (i.e., relapse within 6 months of response) compared with patients experiencing later failure (21.6% vs 9%; *P* = 0.02) [55].

Similarly, in a study of 1698 patients with non-del(5q) lower-risk MDS, Park et al. [62] found that patients experiencing primary ESA failure had a higher risk of progression to AML than those experiencing secondary failure (i.e., relapse after an initial erythroid response; 16.7% vs 8.1%; *P* = 0.001).

### Rationale for the predictive value of sEPO

Despite the clear link between EPO and RBCs, MDS patients with similar hemoglobin levels can have very different sEPO levels [44, 74]. Many, but not all, MDS patients with anemia have elevated sEPO levels, as EPO production is stimulated by hypoxemia [12]. However, despite these high sEPO levels, sufficient RBC production is still not stimulated. Further increasing the concentration of EPO with ESAs is therefore less likely to be effective in patients who already have high sEPO levels. Conversely, elevating sEPO levels with ESAs is more likely to be beneficial among patients with lower levels. Some studies have shown that sEPO levels have a strong inverse correlation with hemoglobin levels in lower-risk MDS patients [35, 75], suggesting that patients with worse anemia (i.e., lower hemoglobin) may have higher levels of sEPO. Patients who are resistant to ESAs have higher sEPO and lower hemoglobin levels than those who respond (Tables 1 and 2), so these factors may be indicative of reduced bone marrow responsiveness.

Several mechanisms for ineffective hematopoiesis in patients with MDS have been discussed in the literature. Spinelli et al. [76] have reported that EPO signaling is affected in MDS patients. They found that EPO failed to activate extracellular signal-regulated kinase (ERK) 1/2 or STAT5 in 64% of cases in CD71 + CD45− cells from patients with MDS [76]. In the same study, in vivo ESA response correlated with in vitro EPO-dependent STAT5 activation in 91% of cases [76]. Frisan et al. [50] have reported that phospho (p)-ERK 1/2 expression—in both the steady state and after EPO stimulation—is defective in cultured MDS erythroblasts. However, Claessens et al. [77] have reported that EPO signaling pathways (STAT5, Akt, and ERK 1/2) are normally activated in MDS erythroid progenitors. Therefore, the role of EPO signaling in patients with MDS is unclear. Methodological differences, including the method used to measure EPO signaling pathway activity, may explain the discrepancy in findings between these studies.

Claessens et al. [77] have also reported that MDS erythroid progenitors have higher apoptosis rates than normal cells—which can be explained by the excess of Fas ligand during the differentiation stage of erythroid progenitors—and that patients with MDS produce less erythroid burst-forming units (BFU-E) than controls. Interestingly, Frisan et al. [50] have reported that responders to ESAs have significantly higher p-ERK 1/2 and BFU-E levels than non-responders (Fig. 1).

Reliable prognostic markers for response can be used to guide treatment options and, hence, improve outcomes. Despite limitations in its assessment, sEPO is an important predictor of response to ESAs with or without G-CSF in patients with lower-risk MDS. Lower sEPO levels (up to 500 IU/L) have thus been included in treatment algorithms for patients with lower-risk MDS to define whether ESAs are indicated [8, 68]. However, in clinical practice, a sEPO cutoff level of 200 IU/L is more likely to be indicative of response, and various scoring systems can be used to further enhance response prediction. For patients who do not respond to ESAs alone, G-CSF can be added; if they are refractory to this combination, other treatment options (e.g., lenalidomide) may be required [8]. Studies of other MDS therapies (e.g., lenalidomide, luspatercept) have also shown that patients with lower sEPO levels are more likely to have a hematologic response. Overall, there is a wealth of evidence that lower sEPO levels are predictive of hematologic response—particularly to ESAs. Further, clinical trials should use sEPO thresholds to ensure more homogeneous cohorts. Previous studies have shown that more than 97% of patients with MDS have sEPO levels < 500 IU/L [62]. Current European guidelines recommend erythropoietin-alpha for patients with sEPO levels < 200 IU/L [78], who represent approximately 86% of patients with MDS [56].
