# Correction to: The prognostic value of serum erythropoietin in patients with lower-risk myelodysplastic syndromes: a review of the literature and expert opinion

**DOI:** 10.1007/s00277-019-03874-w

**Published:** 2019-12-02

**Authors:** Sophie Park, Charikleia Kelaidi, Mathieu Meunier, Nicole Casadevall, Aaron T. Gerds, Uwe Platzbecker

**Affiliations:** 1 CHU Grenoble, Université Grenoble Alpes, Institute for Advanced Biosciences, INSERM U1209, CNRS UMR 5309, CS 10217, 38043 Grenoble, France; 2 Aghia Sophia Children’s Hospital, Athens, Greece; 3 Hôpital Saint-Antoine, Paris, France; 4 Taussig Cancer Institute, Cleveland Clinic, Cleveland, OH USA; 5 Medical Clinic and Policlinic 1, Hematology and Cellular Therapy, Leipzig University Hospital, Leipzig, Germany

**Correction to: Annals of Hematology**

10.1007/s00277-019-03799-4

The article “The prognostic value of serum erythropoietin in patients with lower-risk myelodysplastic syndromes: a review of the literature and expert opinion”, written by Sophie Park, Charikleia Kelaidi, Mathieu Meunier, Nicole Casadevall, Aaron T. Gerds, and Uwe Platzbecker, was originally published electronically in SpringerLink) on October 25, 2019 without Open Access.

With the author(s)’ decision to opt for Open Choice the copyright of the article changed on December 2019 to © The Author(s) 2019 and the article is forthwith distributed under the terms of the Creative Commons Attribution 4.0 International License (http://creativecommons.org/licenses/by/4.0/), which permits use, duplication, adaptation, distribution and reproduction in any medium or format, as long as you give appropriate credit to the original author(s) and the source, provide a link to the Creative Commons license, and indicate if changes were made.

The original article has been corrected.

